# Recent progress and clinical applications of advanced biomaterials in cosmetic surgery

**DOI:** 10.1093/rb/rbad005

**Published:** 2023-02-07

**Authors:** Hairui Li, Xiujuan Xu, Lina Wu, Xi Chen, Haris Akhter, Yixi Wang, Ping Song, Xiaoxia Liao, Zhenyu Zhang, Zhengyong Li, Changchun Zhou, Ying Cen, Hua Ai, Xingdong Zhang

**Affiliations:** Department of Plastic Reconstructive and Aesthetic Surgery, West China Tianfu Hospital, Sichuan University, Chengdu 610213, China; Department of Burn and Plastic Surgery, West China School of Medicine, West China Hospital, Sichuan University, Chengdu 610041, China; National Engineering Research Center for Biomaterials, Sichuan University, Chengdu 610064, China; College of Biomedical Engineering, Sichuan University, Chengdu 610064, China; National Engineering Research Center for Biomaterials, Sichuan University, Chengdu 610064, China; College of Biomedical Engineering, Sichuan University, Chengdu 610064, China; Department of Orthopedic Surgery, West China Hospital, West China School of Medicine, Sichuan University, Chengdu 610041, China; Department of Plastic and Reconstructive Surgery, University of Nebraska Medical Center, Nebraska 68588, USA; Department of Burn and Plastic Surgery, West China School of Medicine, West China Hospital, Sichuan University, Chengdu 610041, China; National Engineering Research Center for Biomaterials, Sichuan University, Chengdu 610064, China; College of Biomedical Engineering, Sichuan University, Chengdu 610064, China; Department of Burn and Plastic Surgery, West China School of Medicine, West China Hospital, Sichuan University, Chengdu 610041, China; Department of Burn and Plastic Surgery, West China School of Medicine, West China Hospital, Sichuan University, Chengdu 610041, China; Department of Plastic Reconstructive and Aesthetic Surgery, West China Tianfu Hospital, Sichuan University, Chengdu 610213, China; Department of Burn and Plastic Surgery, West China School of Medicine, West China Hospital, Sichuan University, Chengdu 610041, China; Department of Plastic Reconstructive and Aesthetic Surgery, West China Tianfu Hospital, Sichuan University, Chengdu 610213, China; National Engineering Research Center for Biomaterials, Sichuan University, Chengdu 610064, China; College of Biomedical Engineering, Sichuan University, Chengdu 610064, China; Department of Burn and Plastic Surgery, West China School of Medicine, West China Hospital, Sichuan University, Chengdu 610041, China; National Engineering Research Center for Biomaterials, Sichuan University, Chengdu 610064, China; College of Biomedical Engineering, Sichuan University, Chengdu 610064, China; National Engineering Research Center for Biomaterials, Sichuan University, Chengdu 610064, China; College of Biomedical Engineering, Sichuan University, Chengdu 610064, China

**Keywords:** applications, biomaterials, cosmetic, soft tissue, scaffolds, regenerative mechanism

## Abstract

Materials of different allogeneic or xenogeneic or autologous origins are widely used as soft-tissue fillers or structural scaffolds in the field of cosmetic surgery, while complications including prosthesis infection, donor site deformity and filler embolization have always been difficult problems for plastic surgeons. The application of novel biomaterials may bring in hopeful solutions for these problems. Recently, some advanced biomaterials, such as regenerative biomaterials can effectively promote the repair of defective tissues, which have been proven to have good therapeutic as well as cosmetic effects in cosmetic surgery. Therefore, biomaterials with active compounds have drawn significant attention for the tissue regeneration of reconstructive and esthetic treatment. Some of these applications have achieved better clinical outcomes than traditional biological materials. This review summarized recent progress and clinical applications of advanced biomaterials in cosmetic surgery.

## Graphical Abstract

**Figure:**
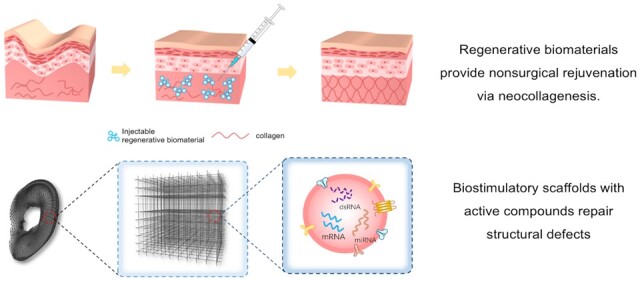

## Introduction

Over the past few decades, the need for cosmetic surgery from the general population has increased dramatically as appearances have become more and more important in the modern society [1]. Rising demand for cosmetic surgery has also been witnessed worldwide [2]. Cosmetic surgeries are primarily elective procedures aiming to improve appearance. Extra cautions should be taken to minimize risks due to the esthetic rather than therapeutic purpose of the treatment. Risk of infection and long-term complications, including prosthesis deformation, displacement and bone absorption, have been reported in rhinoplasty, breast augmentation and additional plastic surgeries [3]. Autogaft such as autologous-fat and rib cartilage, are often scarce and may lead to donor-site complications [4]. All these clinical problems mainly come from the implanted biomaterials or the implants itself.

Recently, advanced biomaterials have been rapidly developed. Advanced biomaterials require their ability to promote tissue regeneration, without additional damage to donor site, risks of prosthetic rejection and long-term infection. For example, injectable biomaterial is a classical biomaterial to provide sustainable and biocompatible effects for facial esthetic injections [5, 6]. The number of repeated injections required is greatly reduced due to the promotion of tissue regeneration, while also reducing the risk of injection-related complications, including the most severe embolism. Vascular embolism, considered as the most serious complication of soft tissue filler injections, may occur less frequently, by promoting tissue regeneration to reduce the number of repeat injections. Ideally, advanced biomaterial would provide an instant filling effect and promote tissue regeneration to provide a long-term filling effect while the biomaterial would degrade over time. However, there has been a mismatch between the ideal filling effect provided by the biomaterial and the filling effect provided by the regenerated tissue for most biomaterials.

There are various advanced biomaterials for plastic surgery. Some have been well verified in clinical practice. Some long-term effects need further study. This review introduced various cosmetic biomaterials that have been successfully applied in clinical practice. Recent progress and clinical applications including injectable advanced biomaterials, additional components in biomaterials for promoting tissues regeneration were summarized [7, 8]. The challenges and future perspective were also discussed. However, translating advanced biomaterial to clinical application is facing great challenges [9]. The interaction between biomaterials and host tissues, the biocompatibility, safety and biodegradability of implanted biomaterials are important issues that need long-term in-depth study.

## Advanced biomaterials for plastic surgery

From the perspective of clinical application, this review more focused on some advanced biomaterials with specific biofunctions, which may help tissue regeneration, biodegradable etc. right now, conventional biomaterials includes hyaluronic acid (HA), poly-l-lactic acid (PLLA), calcium hydroxylapatite (CaHA), bioceramics etc. have been well applied in clinical applications (Table 1).

**Table 1.** Overview of advanced biomaterials in plastic surgery

Clinical administration	Biomaterial	Material type	Clinical usage	Clinical degradation	Properties
Injection	HA	Natural biomaterial: bovine derived Synthetic biomaterial: non-animal derived	Esthetic filler	*Temporary* Pure HA: 3–6 months HA conjugated to another polymer: 6–12 months	Promotes the generation of Type I and Type III Collagen
	CaHA	Synthetic biomaterial	Esthetic filler	*Semipermanent* 12–18 months	Stimulates collagen generation
	PLLA [10]	Synthetic biomaterial	Esthetic filler	*Semipermanent* Up to 2 years	Promotes the generation of Type I and Type III Collagen
	Collagen [11]	Natural biomaterial	Esthetic filler	*Temporary* 9–18 months depending on no. of injections	Replaces collagen environment lost with age
Implant	ADM	Semisynthetic biomaterial	Support Silicone Breast Implants	*Permanent*	Scaffold for host cells to repopulate and revascularize
	Bioceramics	Synthetic biomaterial	Bone defect reconstruction	*Permanent*	Promotes osteogenesis and osteoinductivity

HA, hyaluronic acid; PRP, platelet-rich plasma; PLLA, poly-l-lactic acid; CaHA, calcium hydroxyapatite; ADM, acellular dermla matrices.

### Injectable advanced biomaterials

In the facial area, aging is characterized by the volume loss of soft tissue, especially the atrophy of the skin, due to the shrinkage and redistribution of adipose tissue and the reduction of collagen produced by fibroblasts. Therefore, preventing the loss of subcutaneous fat or the reduction of dermal thickness represents an emerging strategy to combat aging of the face. The use of soft tissue fillers is one of the most common techniques to achieve facial rejuvenation. It increases the volume of soft tissues, flattened wrinkles and filled-up superficial defects immediately. However, side effects are not uncommon with filler injections, including pain, redness, hemorrhage, hematoma, erythema, allergic reactions, vascular events, infection, edema and late-onset adverse events [12].

In recent years, facial anti-aging strategy has developed from volume restoration to pursuing the biostimulatory effects of fillers, that is, the process of promoting the orderly production of cells and tissues by the body through regenerative component and restoring the function of the body tissues, which is the core of the modern anti-aging concept. An ideal filler material is one that stimulates collagen production and promotes extracellular matrix remodeling. Many of the injectable regenerative biomaterials already exist in human tissues, offering good biocompatibility. Biodegradable soft tissue fillers are usually resorbed by the body in 3–24 months [13]. To reduce the risk of complication and improve the acceptability of esthetic injection, it is vital to increase the sustainability of regenerative biomaterial or even make the esthetic effect permanent through regeneration of natural human tissue.

#### Hyaluronic acid

HA is a natural component in connective tissue extracellular matrix. Due to its water-absorbing and hydrophilic characteristics, small mass can occupy large volume with the effect of tissue expansion, at the same time withstand certain pressure, which makes it a filling material that can be applied for different signs of aging by replacing the lost volume [14]. HA has the advantages of being highly versatile and mucoadhesive. Therefore, HA can be used for carrying active factors and drugs [14].

##### Classification of HA

**Figure 1.**
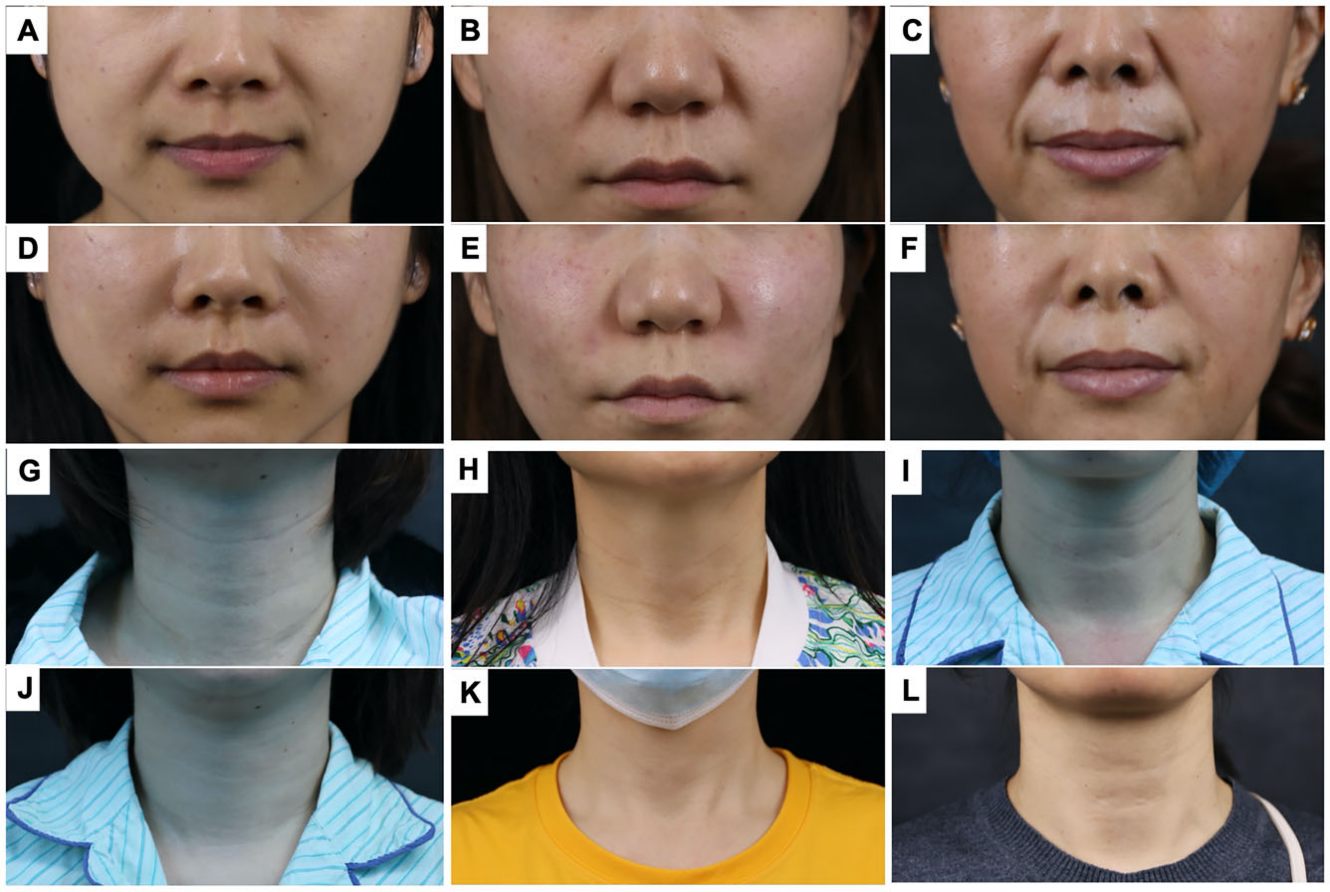
HA used in treatment of nasolabial folds (**A–C**: pretreatment; **D–F**: post-treatment) and neck wrinkles (**G–I**: pretreatment; **J–L**: post-treatment).

In its native form, HA is known as high molecular weight HA, HA with high molecular weight (HMWHA) usually combines with large amount of water, which can then be decomposed to low molecular weight HA (LMWHA) [14]. HMWHA exhibits anti-inflammatory and immunosuppressive properties, while LMWHA exhibits proinflammatory properties [15]. HMWHA can prevent cells from undergoing apoptosis and modulate cell receptors including CD44, which plays a significant role in promoting cell migration and wound healing [16–18]. Both HMWHA and LMWHA have demonstrated strong antioxidant capability [19, 20]. The level of HA cross-linking also affects the cosmetic effect. HA with higher levels of cross-linking provides more robust structural support through promoting type I collagen synthesis [21], while HA with a lower level of cross-linking provides a more elastic and conforming effect due to hydration. Lannitti *et al*. [22] combined cross-linked HA and non-cross-linked HA in the rejuvenation of the skin and achieved satisfactory outcomes. The cross-linked HA is firstly injected to provide structural support, and then non-cross-linked HA is injected to facilitate a more natural appearance similar to the borderline skin (Fig. 1).

##### Regenerative ability

**Figure 2.**
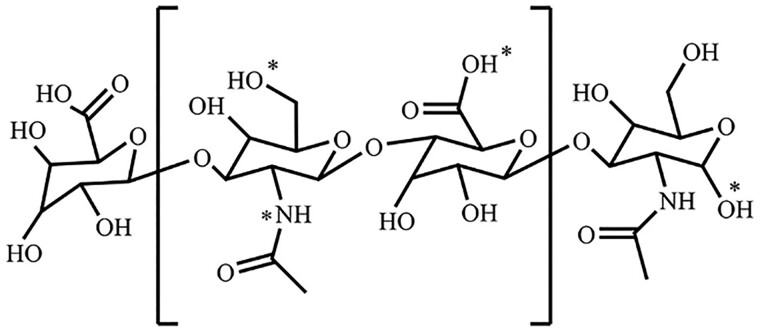
The modification sites (*) of HA [23].

As an injectable biomaterial, HA is commonly used in esthetic surgery. In addition to improving skin hydration and its antioxidant potential [14], HA promotes skin cell regeneration and stimulates dermal fibroblasts to produce collagen [16–18]. Its neocollagenesis mechanism is mainly to promote the formation of new collagen through mechanical stretching and changes in the surrounding structure, thereby inducing the formation of cascades of collagen [14]. Furthermore, HA has a good modification site, so it can be modified to tailor its properties for soft tissue regeneration [14] (Fig. 2).

Ke *et al*. [24] analyzed the underlying inflammation induced by fillers on human skin explants. Cutaneous microdialysis techniques are used to measure levels of interleukin 8 (IL-8), tumor necrosis factor α (TNF-α) and histamine. Injections of fillers have been shown to cause changes of local mechanical stress. This mechanical stress further causes changes in the biochemical, metabolic and secretory patterns of surrounding cells. Wang *et al*. [25] compared forearm skin biopsies in individuals injected with HA or isotonic sodium chloride. Fibroblasts around the injection site have a pronounced elongated mechanically stretched appearance, and immunohistochemistry shows high levels of Type I procollagen. One month after injection, an increase in collagen production was observed and remained elevated for at least 3 months. Scarano *et al*. [6] reported the effect of LMWHA mixed with amino acid on the rejuvenation of the facial skin. The result showed regeneration of Type III reticular collagen and satisfactory clinical outcomes.

##### Complications

Early adverse effects include erythema, ecchymosis, hematoma, oedema, infection, anaphylaxis, vascular infarction, soft tissue necrosis, improper placement and distant spread. Late reactions include infection, nodule and granuloma formation, abscess and HA displacement [26]. Vascular infarction and skin necrosis is rare, but potentially extremely devastating complication. It is the result of untreated vascular damage and can be caused by blockage of arteries or veins. Possible causes are direct damage to the vessel wall, careless injection of injectable fillers into the vessel, or direct compression of fillers on the vessels, resulting in lumen obstruction. Injection-related edema is another possible mechanism that impairs blood flow by applying an external force to the walls of blood vessels. Initial signs and symptoms of impaired blood flow include pain, pallor, discoloration and slow capillary refill. If the artery is occluded, immediate and severe pain occurs, while venous occlusion usually presents with a delayed reticular violet appearance. Due to the biodegradability of the product, the use of hyaluronidase can correct adverse reactions at the moment of injection or after injection [27].

#### Calcium hydroxylapatite

CaHA is a kind of natural mineral existing in the bones which does not require allergy test [13, 28]. CaHA with different morphological structures and particle sizes has different biochemical properties, and the products currently used for cosmetic injections are usually in the form of semi-solid gel. Radiesse^®^ (Merz Pharma GmbH & Co. KGaA, Frankfurt, Germany) contains uniform 25–45 μm CaHA microspheres suspended in a sodium carboxymethylcellulose (CMC) hydrogel matrix. CMC is a gel carrier that has excellent viscosity and elasticity, so it can fill wrinkles immediately after injection. CMC is gradually absorbed by the body after a few weeks of treatment (usually within 8 weeks) [29, 30].

Radiesse has a higher elastic modulus and viscosity compared to HA fillers, providing stronger supporting effect for the injected area. CaHA is FDA-approved for direct injection into the subcutaneous and deep subcutaneous tissues. After injection into the target location, the volume deficit is initially corrected by the gel components and as the gel is gradually absorbed, CaHA microspheres come into contact with the host tissue, activate fibroblasts and promote the regeneration of new tissue, including collagen, proteoglycan and elastin, and other fibrous tissue, which became the main supporting structure 2–3 months after injection [29]. The duration of the lasting effect of CaHA has been reported to range from 12 to 18 months [29, 30].

**Figure 3.**
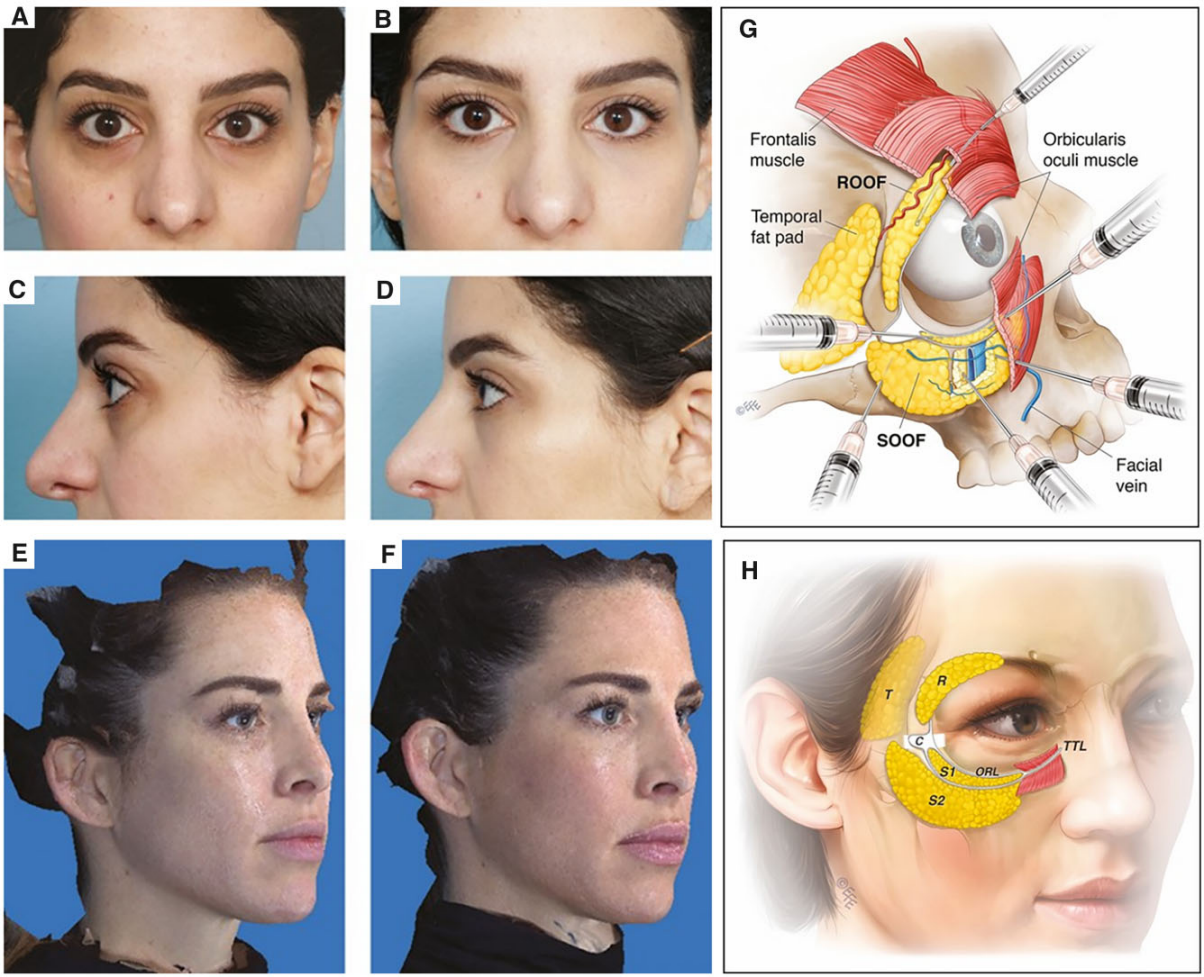
Frontal and lateral views before (**A**, **C**) and 2 weeks after CaHA injection (**B**, **D**). photographs showing right oblique views (**E**) before and 3 months after (**F**) injection of CaHA. The anatomical map (**G**) shows a safe ‘c-angle’ volume augmentation technique relative to the local anatomy [31].

Since CaHA has strong supportive features and is not easily displaced, it is widely used in the treatment of subcutaneous tissue or deep dermal atrophy, including zygomatic area, sub-zygomatic area, suborbital area and nose filling, corner lines, marionette lines, chin lines, mandibular fovea etc. Beer *et al*. [5] reported an 88% satisfaction rate at 6 months and recommended it as a viable option to treat mid-face volume loss, but long-term results were not reported by this study. In a study with 12-month follow-up, CaHA was found to be significantly superior to nonanimal-stabilized HA (NASHA) [31] (Fig. 3).

Complications of Radiesse included cellulitis, tissue necrosis and nodule formation. More serious but uncommon complications include recurrence of shingles, arterial embolism leading to infarction, temporary blindness and oculomotor palsy. Among them, the most worrying complications are vascular damage and tissue necrosis. The interglabellar region is supplied by trochlear artery, resulting in the highest risk of tissue necrosis after filler injection. Nodules may appear when Radiesse is injected into more superficial areas such as the lip mucosa, tear trough and neck lines [32]. Unlike nodules of hyaluronidase-soluble HA-based fillers, CAHA-based filler nodules do not have an established dissolution method and can last for years. Recent case studies have shown that sodium thiosulfate (STS; 250 mg/ml) may be effective in dissolving CAHA nodules [33]. STS is a calcium chelator most commonly used for keratosis, dermatitis or calcifications. One hypothesis is that STS chelates with calcium to form a compound that can be removed through the lymphatic system. Another hypothesis is that it increases the solubility of calcium, thereby reducing the precipitation of calcium in tissues. Since the specific mechanism of STS is still unclear, cautions should be taken by applying conservative dosages. One possible regimen is to inject a small amount of STS into the nodule site (0.1–0.3 ml per palpable nodule) and repeat the injections as necessary at several visits. It is also important to note that it should not be mixed with lidocaine, as this may reduce efficacy.

#### Poly-L-lactic acid

PLLA was first used as a facial filler in 2004 for lipotrophic HIV patients. After that PLLA is more widely used to increase soft tissue volume.

Studies have shown that PLLA is a degradable regenerative biomaterial that promotes the synthesis of collagen in the injected area [34–36]. PLLA particles are between 40 and 63 μm in diameter. This particle size avoids phagocytosis by dermal macrophages and prevents passage through the capillary wall, but is small enough to be easily injected with a needle as fine as 26 gauge. After injection, PLLA filler maintains a certain lactate concentration by continuously releasing lactic acid to promote the host’s own collagen synthesis [34–36]. In rodents, infiltration of lymphocytes and activation of fibroblasts can be observed within extracellular matrix surrounding the microspheres; this is often expected in foreign body reactions [37, 38]. A histological analysis of nasolabial tissue at 12 and 30 months after injection of PLLA showed aggregation of collagen fibers and giant cells [34]. In a clinical study conducted by Stein *et al*. [35], PLLA was injected in the upper arm of 21 patients. After the injection, they found substantial Type III and Type I collagen and upregulated gene expression for their transcripts. At 9 months after the injection, the PLLA was not found under the microscope and was considered completely degraded [35].

In a study including 106 patients, 99.1% of satisfaction was achieved 2 years after injection in the upper face, middle face and lower face regions [39]. Palm *et al*. [40] retrospectively reviewed 130 patients who received PLLA, 75% of the patients rated the treatment effect as good to excellent. It is reported that the effect of PLLA may last as long as 3 years [41]. However, concerns have been raised regarding nodule and papule formation in the early use of PLLA [42], specific consideration of injection technique, the timing of injection sessions and injection volume should be taken.

**Figure 4.**
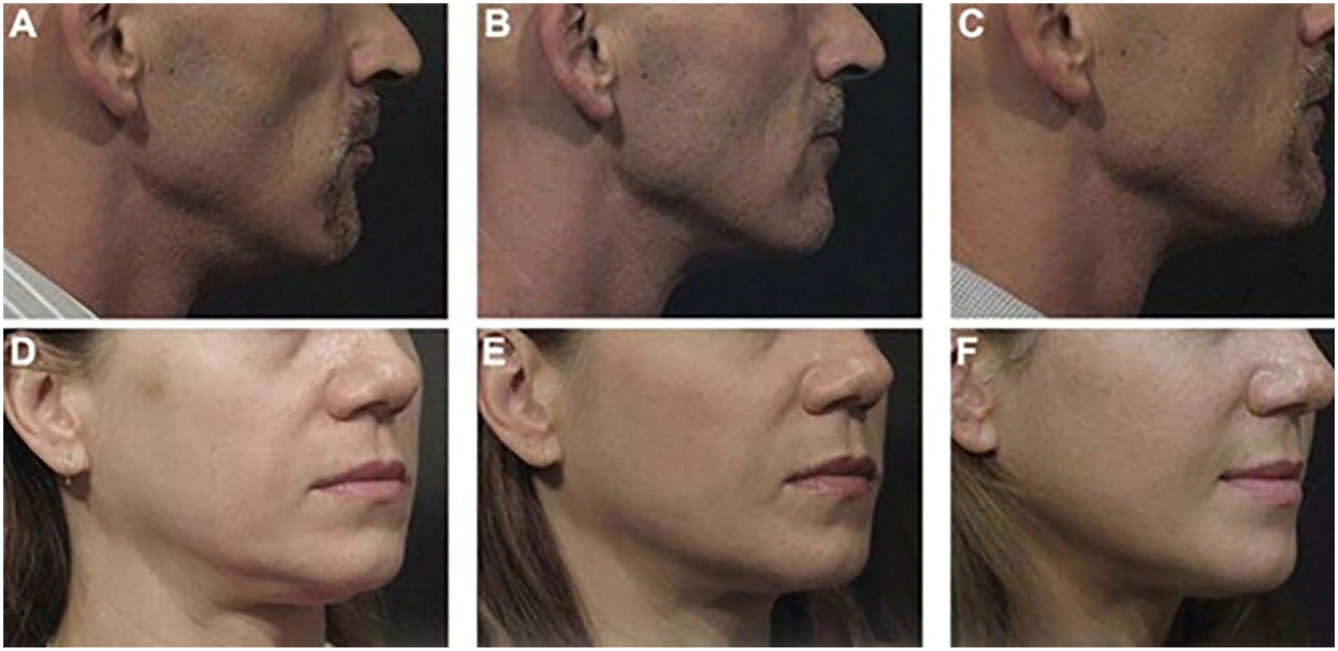
PLLA used in enhanced facial contour [43].

A 48-year-old man presented with facial lipoatrophy (Fig. 4A). The reduction of facial fat magnified bulges caused by muscles and bones. He was injected with three vials of PLLA at a time, three times every 6 weeks (9 vials in total) (Fig. 4B). Figure 4C shows the effect 3 months after the last treatment. Another 42-year-old woman came for aging treatment (Fig. 4D). She was injected with two vials of PLLA at a time and three injections every 4 weeks (six vials in total). Figure 4E shows the effect during treatment, and Fig. 4F was 1 year after last treatment (Fig. 4F) [43].

Hexsel *et al*. [44] describe a new technique that enhances skin and subcutaneous tissue support by injecting small amounts of collagen stimulators into the cheekbones and preauricular area. It gently pulls up the skin, increases the firmness of the skin and delays the normal process of aging facial sagging.

In addition to its classic application in facial area, PLLA is currently used for volume increase, body contouring, sagging skin, scarring and fine line expansion in areas beyond the face such as neck and chest, buttocks, abdomen, arms, thighs, knees and hands. A recent survey reported that hip augmentation (42.4%) is the second most common use of PLLA in the USA, after HIV lipoatrophy (46.8%), which is enough to demonstrate the importance of PLLA in the field of body therapy [45].

Short-term adverse effects of PLLA injection include pain, edema, bleeding, ecchymosis, overcorrection, embolism and localized cellulitis. These side effects usually occur within a few days after the injection and heal on their own within 1–2 weeks [41]. Delayed and persistent nodule is a major complication of PLLA, with an incidence ranging from 5% to more than 40%, but nodule formation can be avoided by proper dilution, placing the product at a deeper anatomical level, minimizing volume in each injection site, and active post-treatment massage (at least five times a day for 5 min for more than 5 days). A retrospective study of more than 100 patients with PLLA injections for cosmetic needs has found that nodules were most likely to appear on the hands (12.5%) and cheeks (7.2%), so cautions should be taken when treating these areas [46]. Avoid injecting PLLA into the lips, as nodules are most likely to form. Treatment options for late-onset subcutaneous nodules include topical steroid injections, systemic steroids, systemic antibiotics, intense pulsed light, 5-fluorouracil, allopurinol and surgical excision.

*Collagen* is a fibrillar protein that naturally exists in many tissues of the human body. It serves a connective role in tissues of the skin, joint and bone [47]. In humans, collagen production decreases with aging, which causes facial wrinkles with a thin dermal layer and flat rete ridges [48]. The common natural source of collagen comes from animals, including bovine and porcine. There are as many as 26 types of collagen characterized by different amino acid sequences and different kinds of cross-linkage [47]. One study reported porcine Type I collagen achieved satisfactory correction of wrinkles in up to 12-month follow-up, comparable to that of bovine collagen [48]. Recent studies have reported that cross-linked collagen may have the ability to prolong the cosmetic effect and show comparable efficacy to that of HA [49, 50]. Delivery methods have also been changing to minimize trauma and discomfort during injection. Sun *et al*. [51] has successfully developed a polyvinylpyrrolidone microneedles system to deliver Type I collagen into human skin.

**Figure 5.**
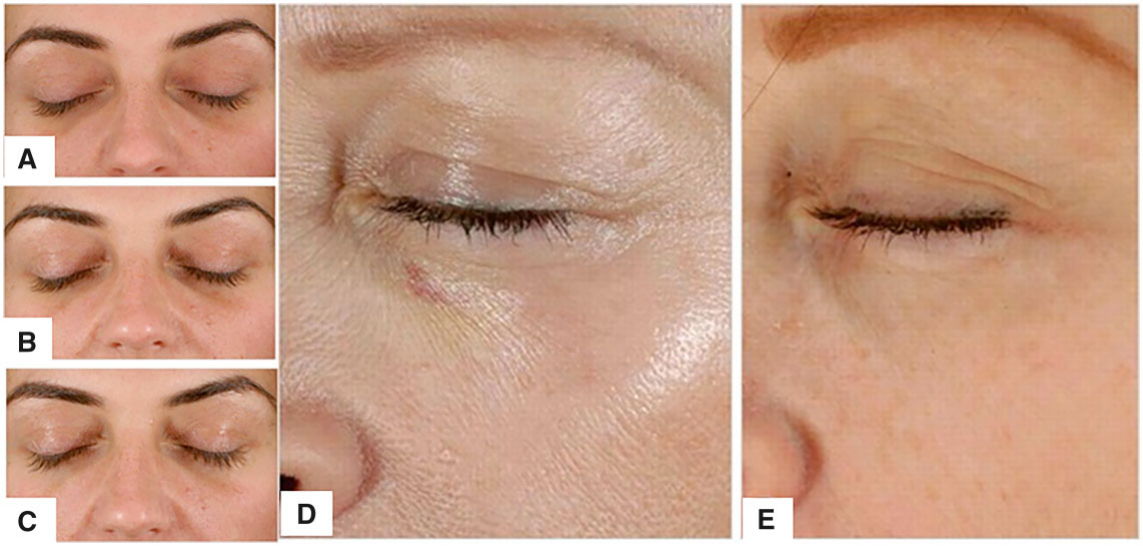
(**A–C**) Pretreatment and post-treatment view of a 31-year-old woman. (**D, E**) Pretreatment and post-treatment view of a 55-year-old woman [52].

Filling the tear trough with collagen is a safe and minimally invasive cosmetic procedure. According to a clinical trial [52], a total of 10 female patients were treated with collagen material for tear trough deformity. All patients had excellent clinical outcomes. No swelling or lumps were observed after treatment. All patients resumed normal work and social activities immediately after treatment. At the 3-month follow-up, patient satisfaction was high (Fig. 5).

### Implanted advanced biomaterials and 3D printing technology

Traditionally, autologous grafts and synthetic permanent implants have been used to repair structural defects or as an augmentation to achieve esthetic effects. However, both autologous grafts and synthetic permanent implants face many challenges. For the autologous graft, the challenges include additional operation conducted at the donor-site, which might lead to donor-site deficit and even further complications. For instance, thoracic deformity can be found in microtia children treated with autologous rib cartilage [53]. Additionally, autologous graft can be insufficient to achieve ideal surgical outcome [54]. For the synthetic permanent implant, challenges include risk of infection, which is always present as long as the implant is within the body. Also, due to the lack of integration with surrounding tissue, the long-term effect may be compromised. Pseudocapsule formation and bone resorption under stress have been reported in breast augmentation, mandible augmentation and rhinoplasty using silicone [3].

For any regenerative biomaterial, the scaffold is considered the basic structure that provides appropriate physical environment for tissue regeneration [55–57]. Various substances can be integrated or added into the scaffold to induce and accelerate the intended biological process (angiogenesis, fibrosis, osteogenesis etc.). The regenerative biomaterial scaffold may be used as a supplement instead of traditional material in plastic and cosmetic surgery. For example, bioceramics including HA, TCP and bioactive glasses have been selected for repairing bone defect. One of the advantages of ceramics constructs is that the ion-rich microenvironment promotes cell proliferation by close cell–cell interaction [58]. It provides structural support for bone tissue and has the potential to interact with the surrounding tissue as well. Recent progress has been made in constructing nano scaled biomimetic scaffolds, including ion-functionalized scaffolds, decellularized extracellular matrix scaffolds [59]. Some of them provide strong structural support as well as physical and bioactive properties, which promote tissue regeneration [60]. The osteogenesis and osteoinductivity can be further enhanced when other materials such as polymers are incorporated. In polymers, polyetherketoneketone has shown promising results in mechanical support and osteointegration in recent studies [61–63]. Hydroxyapatite combined with osteogenesis induction materials shows promising outcome in treating bone defects. Hydroxyapatite provides structural support for about 1-2 years before degradation while the osteogenesis process gradually fills the defected area.

The complexity of structure and function in the craniofacial areas makes reconstructing challenging. Extensive research has been conducted on the use of regenerative biomaterials and 3D printing technologies in the reconstruction of bone and cartilage defects in specific region [64]. Three-dimensional (3D) printing has the capability to remodel the regenerative biomaterial into an ideal shape based on personalized clinical needs. Porosity, elasticity, surface morphology, and other physical properties can be modified by integrating different material and printing techniques. A recent study has also shown the possibility of printing cells into a construct [65]. Inzana *et al*. [66] printed calcium phosphate constructure while incorporating a collagen coating, making the implant osteoconductive, biodegradable, and with a stable 3D structure. Another study using inkjet technique integrated other osteoconductive materials, including BMP-2, into micro-porous scaffolds and observed tissue formation [67]. Integrating 3D printing and regenerative biomaterial may provide a personalized, stable and self-regenerative biomaterial with added pro-regeneration ingredients.

Few attempts have been made in the clinical application of 3D printed regenerative biomaterial to reconstruct craniofacial defects. Brie *et al*. [68] used HA and resin as material to produce 3D-printed implants and treated 8 patients with craniofacial defects. In 12-month follow-up, no major complications were observed, and the cosmetic effect were considered satisfactory. Staffa *et al*. [69] developed fully biodegradable implant using high porosity HA, which achieved satisfactory esthetic result with no prosthesis fragmentation note in 12–79 months follow up. Hikita *et al*. [70] used α-TCP instead of HA or β-TCP as the printing material since there is no need for acid-based binders or polymer solution with α-TCP, therefore, it may provide better biodegradability.

## Additional components for promoting tissues regeneration

On the basis of ensuring biocompatibility and safety, advanced biomaterials can be compounded with some other additional components. These additional components can generate some synergistic effects to promote tissue repair and regeneration. Key components including growth factors, platelet rich plasma (PRP), stem cells, extracellular vesicles (EVs) and growth factors, which can be integrated into biomaterials for further enhanced biofunctions.

### Platelet rich plasma

Activated platelet release a variety of growth factors and cytokines, which mediate inflammation, angiogenesis and synthesis of ECM. Platelet-derived growth factors and fibroblast growth factors have had positive influence on inflammation response, granulation tissue formation and remodeling process. Vascular endothelial growth factor derived from PRP have been proved shown an important role in promoting the formation of skin capillaries, and it is conducive to skin repair and regeneration [71]. One study combined PRP and grafting biomaterial, and it showed superior osteogenesis compared with graft alone [72]. As endogenous signaling molecules, growth factors play a crucial role in regulating tissue regeneration.

In esthetic dermatology, collagen stimulation is the main purpose for using PRP. Studies have shown improved skin color and texture with PRP injection through intradermal or subdermal injection. In 5 months after three injections of PRP into the wrinkles of the face, significant improvement was found regarding general appearance, skin firmness and correction of wrinkles [73]. When combined with micron-needling, PRP effectively improved the appearance of acne scars [74]. PRP should have a concentration of platelets four to seven times above the physiologic concentration. However, the relationship between PRP concentration and its efficacy is not fully investigated [75–78].

### Stem cell

Studies have shown that biomaterial combined stem cell therapy could promote muscle and bone regeneration [79]. Stem cells have the potential to differentiate into many esthetic related tissues. Stem cells are conducive to the differentiation of fibroblasts and endothelial cells, which is good for promoting muscle tissue regeneration. Biomaterials may regulate the microenvironment of the stem cell and promote proliferation and differentiation [80]. Biomaterial provides the surrounding environment to support specific cellular functions. Previous studies reported polymeric materials, including Collagen I, supported myogenesis [81]. The proteins, such as fibrin would promote myogenesis too. Some positive results have been observed in animal models of muscle trauma [82, 83].

### Extracellular vesicles

EVs have attracted considerable attention over the past few years. EVs are small particles of nanoscale vesicles with lipid membranes secreted by cells, which are shed by almost all types of cells *in vitro* and *in vivo*. The classifications of EVs mainly include apoptotic bodies, microvesicles and exosomes. They are consisted of phospholipid bilayers incorporating several different surface and membrane proteins. Furthermore, EVs can carry microRNA, proteins and resistance genes. EVs are cellular information disseminators which can specifically transfer biological information between donor and recipient cells. Therefore, EVs can be delivered by some biomaterials to treat diseases.

**Figure 6.**
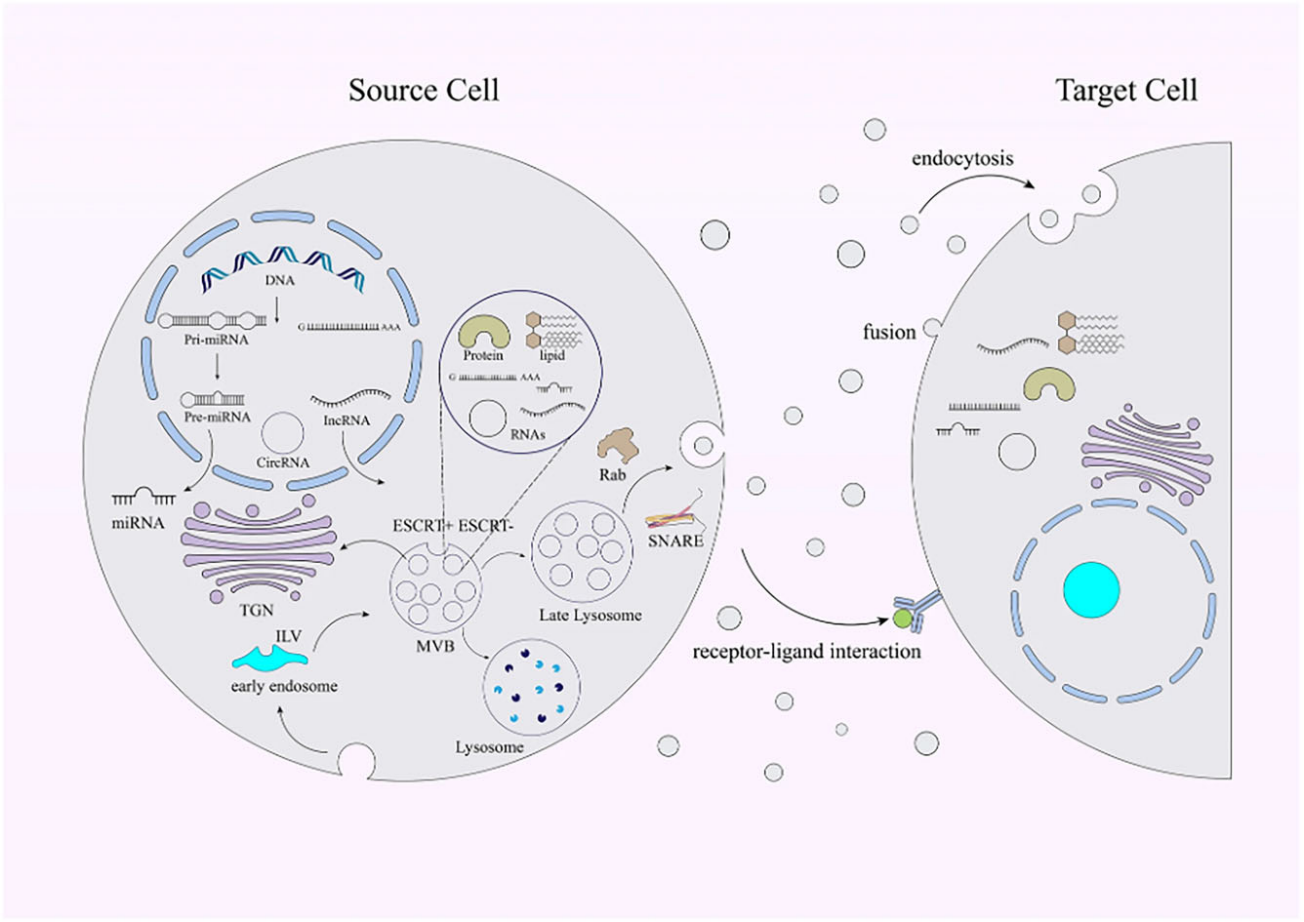
Schematic illustration of the biogenesis, compositions and also release of the EVs [84].

One of the advantages of EVs is that they exhibit low immunogenicity when used autologously. Therefore, EVs potentially have limited side effects. The size and lipid membrane composition of EVs allow them to fuse with target cells while avoiding degradation easily. In addition, EVs avoid inherent toxicity compared to synthetic nanoparticles (Fig. 6).

### Growth factor

Tissue regeneration aims to achieve functional recovery after injury by creating an environment that enables self-repair. In order to achieve tissue regeneration, it is necessary to introduce exogenous cells, biomaterial scaffolds and bioactive molecules into the tissue. Growth factors play important roles in directing regeneration pathways among these active ingredients. Growth factors belong to a new class of polypeptide hormones. These polypeptides can stimulate DNA synthesis and mitosis in cells. Growth factors have been isolated from platelets, submaxillary gland, pituitary gland, brain and cultured cells *in vitro*.

Exogenous growth factors are now widely used in trauma and wound healing treatment, such as epidermal growth factor and fibroblast growth factor, which are the main kinds of growth factors applied. Usually, biomaterials such as hydrogels are used as carriers, along with growth factors to be injected subcutaneously or into muscles, which can promote tissue repair. The deficiency of growth factors has been proven to be associated with delayed wound healing [85], which makes applying growth factors a potential treatment option. However, the effect of the therapeutic application of growth factors is limited because of the low stability and limited absorption. It is difficult to send enough active and stable growth factors to the designated area and maintain a specific concentration in the area. Delivery systems based on biomaterials may help us overcome this difficulty [86]. PLGA, alginate microspheres, HA and collagen have been used as the drug delivery systems either separately or combined [87–90]. Recently, developments have been reported in creating a porous topology of natural ECM using micro nanofibers electrospinning technique [91, 92].

## Challenges and future perspective

On the premise of safety and minimizing complications, maximizing the effect of treatment is the unremitting pursuit for plastic surgery. Take ear reconstruction as an example, microtia is a congenital malformation of the external ear, which results in abnormal appearance and loss of function. Autologous rib cartilage remained the primary option for microtia reconstruction. However, scarcity of the cartilage, the influence of thoracic growth and the skill required for a surgeon to carve the complex 3D structure of the external ear remained some problems [4]. The utilization of regenerative biomaterial in this field solved the problem of source scarcity. More artificial synthetic biomaterials, instead of being taken from patients themselves, will be an important direction of development in cosmetic surgery field. In an ideal scenario, there is a dynamic balance between the beauty and the safe. Security, minimally invasive and permanent repair are the best choices. However, at present, how to accurately control compatibility, biodegradation, retention time and other aspects remains to be explored.

On the other hand, some challenges still need to be concerned. Some traditional biomaterials, such as CaHA, PMMA and PLLA for cosmetic injection, there are still many clinical cases of failure reported. Some permanent or semi-permanent biomaterials take years, or even decades, to degrade *in vivo*. Which may cause multiple complications after subcutaneous injection cases, including subcutaneous lesions, erythema, granuloma etc. The improvement of the biocompatibility, biosafety and biodegradability of these materials is a great challenge. In addition, different countries have different approval and access thresholds. How to effectively evaluate the safety of regulatory cosmetic surgery biomaterials is also another major challenge. These problems should not be ignored.

## Conclusions

Although faced with various challenges, regenerative biomaterial provides promising treatments in the field of plastic surgery. Additional investigations should be focused on translating the research achievements to clinical practice in some of these promising regenerative biomaterials while ensuring biocompatibility and safety. The quality of ideal regenerative biomaterial includes: (i) lasting effect, (ii) safety, (iii) degradable, (iv) regenerative, (v) easy to remodel and (vi) dynamic balance between degradation and regeneration. Therefore, the regenerative biomaterial is gradually replaced by the regenerative tissue while sustaining satisfactory surgical effect throughout the treatment.
